# HIF‐Mediated Fructose Metabolism and Disease Progression in the Cardiovascular‐Kidney‐Metabolic Syndrome

**DOI:** 10.1002/cph4.70033

**Published:** 2025-08-11

**Authors:** David Mathew, Sean Davidson, Derek Yellon

**Affiliations:** ^1^ The Hatter Cardiovascular Institute University College London London UK

**Keywords:** fructose, HIF, hypoxia, Ketohexokinase

## Abstract

The ‘Cardiovascular‐Kidney‐Metabolic Syndrome’ which is characterized by multi‐organ dysfunction ultimately resulting in adverse cardiac outcomes, serves to highlight the importance of organ crosstalk in pathophysiology. The cellular metabolism of fructose, regulated by Ketohexokinase‐C with associated inflammatory sequelae, is mechanistically linked with each component of this clinical entity. Fructose metabolism is confined to the Kidney, Liver, and Small Intestine under normal physiological conditions; however, in the context of ischaemia, HIF‐1α induces cardiac expression of Ketohexokinase‐C with consequent organ hypertrophy and dysfunction. This adverse effect of cardiac HIF‐1α accumulation raises concerns over the potential pleiotropic effects of the ‘HIF stabilizing’ inhibitors of Prolyl Hydroxylase currently entering clinical practice for the treatment of anemia in Chronic Kidney Disease, particularly given the increased cardiovascular mortality observed in this patient group. We suggest that pleiotropic effects of ‘HIF stabilization’ on cardiac physiology warrant investigation and, furthermore, that pharmacological inhibition of Ketohexokinase‐C, and therefore fructose metabolism, represents an opportunity to improve cardiac outcomes in the Cardiovascular‐Kidney‐Metabolic Syndrome.

## Graphical abstract

Under normoxic conditions in cardiomyocytes, Hypoxia Inducible Factor‐1 alfa (HIF‐1α) is degraded by Prolyl Hydroxylases and Ketohexokinase‐A (KHK‐A) is expressed. Hypoxia results in stabilization of HIF‐1α with consequent induction of Ketohexokinase‐C (KHK‐C) expression via Splicing Factor 3B1 (SF3B1). The subsequent metabolism of fructose by KHK‐C results in inflammation and organ dysfunction. Pharmacological stabilization of HIF‐1α in the context of Chronic Kidney Disease may induce a similar cardiac phenotype. Pharmacological inhibition of KHK‐C may improve cardiac outcomes in the ‘Cardiovascular‐Kidney‐Metabolic Syndrome’.

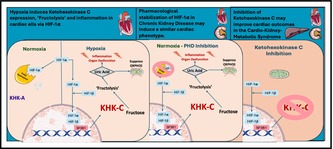

**Abbreviations**
CKD: chronic kidney disease
CKMS: cardiovascular‐kidney‐metabolic syndrome
EPO: erythropoietin
HIF: hypoxia inducible factor
KHK‐C: Ketohexokinase‐C
NAFLD: non‐alcoholic fatty liver disease
SF3B1: splicing factor 3B subunit 1

Investigation of the pathophysiology impacting cardiac, renal, and metabolic health outcomes has identified numerous common processes between them. This highlights the fact that there are important connections between these organs, despite them typically having been studied only in isolation. The new term ‘Cardiovascular–Kidney‐Metabolic Syndrome’ (CKMS) describes the clinical presentation of a multisystem disorder, the common end point of which is adverse cardiovascular outcomes (Ndumele et al. 2023; Pearce et al. 2025). The cellular metabolism of fructose, with associated inflammatory sequelae and organ dysfunction, represents one process underlying this disorder. However, this process is incompletely understood despite it having implications both for disease progression in CKMS as well as the choice of treatment modality for example, the use of stabilizers of hypoxia inducible factor (HIF) in Chronic Kidney Disease (CKD).

Epidemiological and mechanistic studies link excess fructose consumption to each component of the CKMS (Sun et al. 2023). This is due in part to the cellular metabolism of fructose (Figure 1), which bypasses the regulatory checkpoint of glycolysis thanks to a lack of negative feedback inhibition on the rate limiting ‘Fructolytic’ enzyme, Ketohexokinase‐C (KHK‐C). This results in intracellular phosphate depletion, uric acid generation, and downstream inflammation via multiple mechanisms (Cirillo et al. 2009). In addition, conditions of ‘physiological stress’, that is, hypertonicity, hypoxia, and hyperglycaemia, which are abundant in CKMS, stimulate activation of the ‘Polyol Pathway’, which converts cytosolic glucose to fructose. This fructose, produced *de novo*, could subsequently be robustly phosphorylated unimpeded by negative feedback, with resultant inflammation and organ dysfunction. This milieu would be further exacerbated by excessive dietary fructose intake and in the context of diabetes, where serum fructose concentrations are elevated (Kawasaki et al. 2002).

**FIGURE 1:**
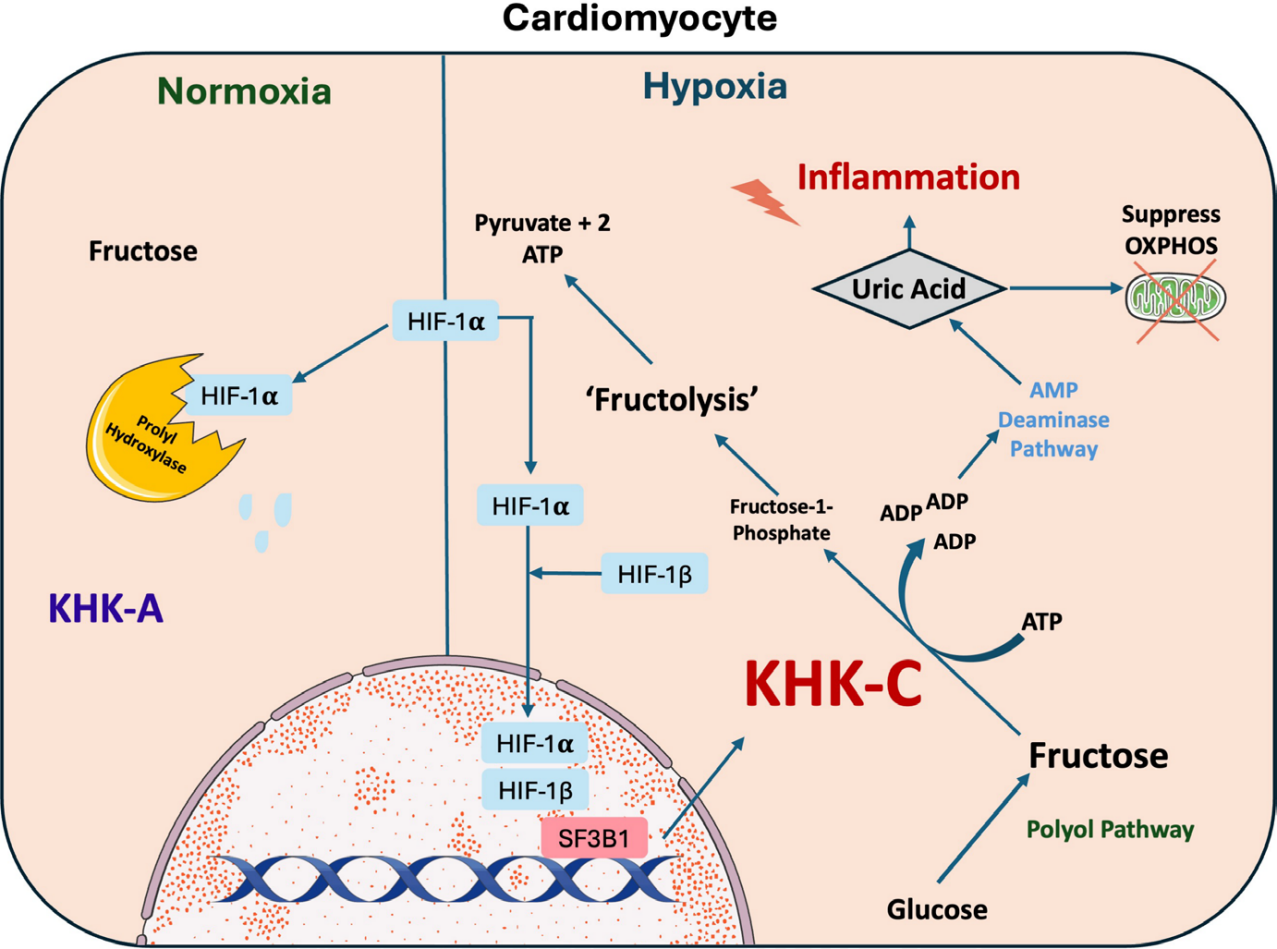
HIF‐1α Mediated isoform switch of Ketohexokinase and subsequent fructose metabolism. During normoxia cardiomyocytes express KHK‐A which has a low affinity for fructose metabolism and HIF1α is degraded by prolyl hydroxylase. In the context of hypoxia HIF‐1α heterodimerizes with HIF1β and translocates to the nucleus inducing an isoform switch to KHK‐C via SF3B1. In addition, hypoxia activates the Polyol Pathway resulting in the production of *de novo* fructose. This fructose could then be robustly phosphorylated by KHK‐C, which has no negative feedback mechanism ultimately resulting in pyruvate and a net gain of two ATP. Resultant phosphate depletion activates the AMP Deaminase Pathway with consequent uric acid production and associated inflammation. Uric Acid suppresses aconitase resulting in suppression of oxidative phosphorylation and a shift towards glycolysis.

Members of the HIF family of transcription factors, specifically HIF‐1α, are important modulators of fructose metabolism in cardiomyocytes (Mirtschink et al. 2015). The heart does not metabolize fructose at rest; however, this is ‘activated’ in response to hypoxia, as HIF‐1α mediates an isoform switch from the inactive KHK‐A to the physiologically active KHK‐C via Splicing Factor 3B Subunit 1 (SF3B1) (Figure 1) (Mirtschink et al. 2015). In animal models of chronic disease, this response induces cardiac hypertrophy and dysfunction, which is abolished by deletion of KHK. In addition, human myocardial biopsies from patients with chronic ischaemic pathology (Aortic Stenosis and Hypertrophic Cardiomyopathy) demonstrate increased HIF‐1α, SF3B1, and KHK‐C mRNA as well as protein. These findings directly implicate fructose metabolism in the maladaptive response to chronic cardiac ischaemia (Mirtschink et al. 2015).

The role of fructose metabolism in cardiac dysfunction and its modulation by HIF‐1α has implications for CKD, as a class of ‘HIF stabilizing’ compounds has recently entered clinical practice. These compounds, e.g., Roxadustat, are intended as an alternative to existing therapies and exploit the effect of HIF‐1α to increase Erythropoietin (EPO) expression, thereby compensating for the lack of EPO and the consequent anemia associated with CKD. However, the pleiotropic nature of HIF stabilizers has raised concerns about their use in CKD; one potential unintended consequence being the activation of cardiac fructose metabolism and associated deleterious effects. Given that CKD itself confers a greatly increased risk of cardiac mortality (Matsushita et al. 2010), due consideration and investigation should be given to this additional risk. Individual pre‐clinical models have identified adverse cardiac outcomes in the context of chronic HIF stabilization, although to date a link with fructose metabolism has not been investigated, and an increase in cardiovascular mortality has not been observed in clinical trials, possibly due to the limited duration of treatment (Moslehi et al. 2010; Zheng et al. 2020).

Aside from considerations related to treatment of CKD associated anemia, cardiac fructose metabolism induced by physiological stress (as observed in CKMS) presents an interesting therapeutic target in the form of pharmacological inhibition of KHK‐C. Such inhibition would abrogate cardiac fructose metabolism entirely, thereby reducing inflammation, hypertrophy, and organ dysfunction.

This approach has provided encouraging results in the pre‐clinical setting with regard to renal injury, where fructose metabolism and associated inflammation are also implicated both acutely and chronically. In knockout models, KHK A/C null mice demonstrate improved renal outcomes in response to ischaemic Acute Kidney Injury, an effect which was replicated pharmacologically with the flavone Luteolin, an effective inhibitor of KHK A/C (Andres‐Hernando et al. 2017). Furthermore, a potent and reversible inhibitor of KHK, ‘PF‐0683591’ has successfully decreased hepatic steatosis, uric acid, and inflammatory markers in a randomized phase 2 trial of human subjects with Non‐Alcoholic Fatty Liver Disease (NAFLD) (Kazierad et al. 2021).

In addition to the liver and kidney specific benefits of KHK inhibition, we speculate that an effective treatment strategy would be to target cardiac fructose metabolism in CKMS via KHK inhibition. The cardiac metabolism of fructose is a maladaptive physiological response to the CKMS, not present in the healthy heart, which contributes to adverse cardiac outcomes, the common end point of this multisystem disease. As such, an effective inhibitor of cardiac KHK‐A/C provides an interesting and as yet unexplored potential for clinical benefit in CKMS. The aforementioned ‘PF‐0683591’ could be considered for this purpose, given that it has demonstrated adequate cardiac tissue penetration on pharmacokinetic characterization (Guodong et al. 2023). Autosomal recessive loss of function of Ketohexokinase has been described in humans and is termed ‘Essential Fructosuria’; however, the rarity of this condition means that cardiac outcomes have not been conclusively investigated (Laron 1961).

We also suggest that further work to investigate the pleiotropic effects of HIF‐stabilizers, for example, Roxadustat, on cardiac fructose metabolism and adverse consequences should be pursued, given that the use of these agents in CKMS.

## Conflicts of Interest

The authors declare no conflicts of interest.

## Data Availability

Data sharing is not applicable to this article as no new data were created or analyzed in this study.
